# Mammary Carcinoma Cell Derived Cyclooxygenase 2 Suppresses Tumor Immune Surveillance by Enhancing Intratumoral Immune Checkpoint Activity

**DOI:** 10.1186/bcr3469

**Published:** 2013-09-03

**Authors:** Nune Markosyan, Edward P Chen, Rebecca A Evans, Victoire Ndong, Robert H Vonderheide, Emer M Smyth

**Affiliations:** 1 Institute for Translational Medicine and Therapeutics, Smilow Center for Translational Research, 3400 Civic Center Blvd, Philadelphia, PA 19104, USA; 2 Abramson Family Cancer Research Institute, 421 Curie Blvd, Philadelphia, PA 19104, USA; 3 Department of Medicine, Perelman School of Medicine at the University of Pennsylvania, 415 Curie Blvd, Philadelphia, PA 19104, USA

**Keywords:** breast cancer, immune modulation, tumor microenvironment, cytotoxic immune cells, PD-L1

## Abstract

**Introduction:**

Systemic inhibition of the inflammatory enzyme cyclooxygenase (COX) 2 decreases the risk of breast cancer and its recurrence. However, the biology of COX-2 in the multicellular tumor microenvironment is poorly defined.

**Methods:**

Mammary tumor onset and multiplicity were examined in ErbB2 transgenic mice that were deficient in mammary epithelial cell COX-2 (COX-2^MEC^KO) compared to wild type (WT) mice.

Tumors were analyzed, by real time PCR, immune-staining and flow cytometry, for proliferation, apoptosis, angiogenesis and immune microenvironment. Lentiviral shRNA delivery was used to knock down (KD) COX-2 in ErbB2-transformed mouse breast cancer cells (COX-2KD), and growth as orthotopic tumors was examined in syngenic recipient mice, with or without depletion of CD8^+ ^immune cells.

**Results:**

Mammary tumor onset was delayed, and multiplicity halved, in COX-2^MEC^KO mice compared to WT. COX-2^MEC^KO tumors showed decreased expression of Ki67, a proliferation marker, as well as reduced VEGFA, its receptor VEGFR2, endothelial NOS and the vascular endothelial marker CD31, indicating reduced tumor vascularization. COX-2^MEC^KO tumors contained more CD4^+ ^T helper (T_h_) cells and CD8^+ ^cytotoxic immune cells (CTL) consistent with increased immune surveillance. The ratio of T_h _markers Tbet (T_h_1) to GATA3 (T_h_2) was higher, and levels of Retnla, a M2 macrophage marker, lower, in COX-2^MEC^KO tumor infiltrating leukocytes compared to WT, suggesting a prevalence of pro-immune T_h_1 over immune suppressive T_h_2 lymphocytes, and reduced macrophage polarization to the immune suppressive M2 phenotype. Enhanced immune surveillance in COX-2^MEC^KO tumors was coincident with increased intratumoral CXCL9, a T cell chemoattractant, and decreased expression of T lymphocyte co-inhibitory receptors CTLA4 and PD-1, as well as PD-L1, the ligand for PD-1. PD-L1 was also decreased in IFNγ-treated COX-2KD mouse mammary cancer cells *in vitro *and, compared to control cells, growth of COX-2KD cells as orthotopic tumors in immune competent mice was markedly suppressed. However, robust growth of COX-2KD tumor cells was evident when recipients were depleted of CD8^+ ^cells.

**Conclusions:**

The data strongly support that, in addition to its angiogenic function, tumor cell COX-2 suppresses intratumoral cytotoxic CD8^+ ^immune cell function, possibly through upregulation of immune checkpoints, thereby contributing to tumor immune escape. COX-2 inhibition may be clinically useful to augment breast cancer immunotherapy.

## Introduction

The inducible form of cyclooxygenase (COX), COX-2, and one of its pro-inflammatory products, prostaglandin (PG) E_2_, are strongly implicated in a range of human cancers including breast cancer [1,2]. Global deletion or pharmacological inhibition of COX-2 suppressed tumorigenesis in mice [3,4] and humans [5]. PGE_2 _signals through multiple pro-tumor pathways, including PI3K/AKT, RAS-MAPK/ERK and Gs-axin-ß-catenin signaling, to increase tumor cell survival, inhibit apoptosis, increase cancer cell motility, stimulate angiogenesis and inhibit immune surveillance [6].

In the last decade it has become clear that the tumor microenvironment is critical for tumors to survive and progress. In addition to vascular supply, the interplay of tumor cells with non-malignant cells in the stroma provides growth, survival and motility advantages. A central part of the tumor microenvironment is infiltration of immune cells, which can positively or negatively influence tumor progression depending on their differentiation [7,8]. Tumor rejection is favored through T helper 1 (T_h_1)-derived cytokines that drive antigen-presenting and pro-immune M1 macrophage functions, and by the direct tumoricidal actions of CD8^+ ^cytotoxic T lymphocytes (CTLs) and natural killer (NK) cells [6]. However, as tumors progress, soluble mediators and cellular interactions are thought to reprogram immune cells to type 2 functions so that T_h_2 lymphocyte-derived cytokines polarize macrophages to the M2 phenotype to suppress CTLs, promote angiogenesis and support tumor growth [6,9]. In breast cancer, poor prognosis is associated with elevated T_h_2 lymphocytes and tumor-associated macrophages (TAM), while T_h_1 lymphocytes, CTLs and NKs correlate with enhanced survival [8,10], raising intense interest in therapeutic approaches to modify the tumor immune microenvironment. COX-2-derived PGE_2 _has emerged as a tumor-derived mediator that contributes to development of immune tolerance [11-13]. Several studies report the association of tumor COX-2 with infiltrating T cells, dendritic cells, myeloid derived suppressor cells and macrophages [14-17], while PGE_2 _has been linked to immune suppression in hepatocellular carcinoma [18], lung [19], ovarian [20] and breast [14,15] cancers. The mechanisms through which COX-2/PGE_2 _suppress immune function are poorly defined; however, PGE_2 _suppressed the ability of mature CTLs to kill murine plasmocytoma cells [21] and inhibited T_h_1 generation of interferon γ (IFNγ) [22,23], a cytokine that is critical to sustain anti-tumor immune function [6,24].

We reported that selective deletion of mammary epithelial cell (MEC) COX-2 (COX-2^MEC^KO) delayed carcinogen-induced mammary tumor onset coincident with enhanced markers of anti-tumor type 1 immunity [17]. Chemical carcinogens are generally not, however, considered significant in human breast cancer etiology; therefore, in the current study, we investigated the role of tumor cell COX-2-derived mediators in ErbB2 (HER-2/neu)-induced mammary tumorigenesis. ERBB2 gene amplification or overexpression of the HER-2 protein has been identified in 25% to 34% of human breast cancers [25,26]. ErBb2 mouse models show remarkable morphological resemblance to some forms of human breast cancer and accurately recapitulate the hallmark changes associated with the early stages of human breast cancer [27]. In COX-2^MEC^KO mice transgenic for an activated ErbB2 mutant, we determined delayed tumor onset and reduced tumor multiplicity, as well as reduced tumor vascularization, compared to wild type (WT). Deletion of COX-2 in tumor cells also significantly impaired maintenance of pro-tumorigenic lymphoid and myeloid cell functions thereby facilitating enhanced immune surveillance.

## Methods

### Mice and tumor tissue collection

All procedures were conducted in accordance with National Institutes of Health regulations and were approved by the Institutional Animal Care and Use Committee of the University of Pennsylvania.

Floxed COX-2 mice, generated by flanking the COX-2 gene between introns 5 and 8 with loxP sites (COX-2^flox/flox^), were backcrossed fully (>9 generations) onto an FVB background and are denoted as wild type (WT) mice. COX-2^flox/flox ^mice were crossed with FVB mice expressing Cre-recombinase under control of the mouse mammary tumor virus (mmtv) promoter (Cre^mmtv^), which is used widely to target transgene expression to MEC. The resulting mice were termed COX-2^MEC^KO and their characterization is described in our previous work [17]. WT and COX-2^MEC^KO were crossed with mice transgenic for the ErbB2 (HER2/c-neu) oncogene carrying Eactivating Val^664 ^to Glu^664 ^mutation (Jackson Laboratory, Bar Harbor, ME, USA), also expressed under the control of mmtv promoter. Genotype verification was performed by conventional PCR using primers listed in Table 1.

**Table 1:** Genotyping primer sequences

Gene	Forward (F) and Reverse (R) primer sequences
COX-2	F:TGA GGC AGA AAG AGG TCC AGC CTT
	R:ACC AAT ACT AGC TCA ATA AGT GAC
Cre^mmtv ^	F:TCG ATG CAA CGA GTG ATG AGG
	R:ACG AAC CTG GTC GAA ATC AGT
Erbb2	F:GGACATCCAGGAAGTTCAGGGTTAC
	R:ACAGGAGCCAGTTGGTTATTCTTG

Mice were palpated weekly and considered tumor bearing if a palpable mammary mass persisted for more than one week. On necropsy, tumors were counted and isolated from surrounding tissues, after which they were either frozen and stored at -80°C for RNA extraction or fixed in Prefer (Anatech, Battle Creek, MI, USA) overnight and paraffin embedded or digested to obtain single cell suspension for flow cytometry and microbead separation. For tissue digestion, tumors were washed with (D)MEM/F12 + 5% fetal bovine serum (FBS) + gentamycin 50 mg/ml, minced and placed in digestion buffer consisting of 9 parts of wash buffer +1 part collagenase/hyaluronidase (StemCell Technologies, Vancouver, BC Canada). After two hours shaking at 37°C, the suspensions were centrifuged at 1,000 rpm for five minutes. Pellets were washed and treated with red cell lysis buffer (1 part HBSS+2%FBS + 3 parts NH_4_Cl) and then with Trypsin-ethylenediaminetetraacetic acid (EDTA) 0.25% (Gibco, Grand Island, NY, USA), followed by Dispase and DNase (StemCell Technologies). Thereafter, cell pellets were passed through a 40 μm cell strainer, counted and re-suspended either in fluorescence-activated cell sorting (FACS) buffer for flow cytometry (description below) or in degassed MACS buffer (PBS + 0.5% BSA + 2 mM EDTA) for positive selection of CD45^+ ^cells using CD45-microbeads (Miltenyi Biotec, Auburn, CA, USA) according to the manufacturer's instructions.

### NAF mammary tumor cell line culture, transduction, and treatments

The NAF tumor cell line, which was generated from mammary tumors of ErbB2-transgenic mice, was kindly provided by Dr. Lewis Chodosh (University of Pennsylvania). NAF were cultured in (D)MEM medium containing 10% FBS, 1% L-glutamine and 1% penicillin/streptomycin. For viral transduction, 15,000 cells/well were plated on 96-well plates. Mission plKO.1-puro Transduction Lentiviral Particles (20 μl), carrying either non-target control small hairpin RNA (shRNA) or COX-2 shRNA (Sigma, St. Louis, MO, USA), at 1 × 10^7 ^TU/ml were added to the wells with 8 μg/ml protamine sulfate. After 18 hours, lentiviral particles were removed and cells kept in medium containing 2 μg/ml puromycin (Sigma) to select for transduced cells. COX-2 knock down in COX-2 shRNA transduced NAF cells (NAF COX-2KD) compared to non-target shRNA transduced cells (NAF nt) was verified by Q-PCR. Cells were serum starved for 24 hours and then treated with 10 ng/ml IFNγ(PeproTech, Rocky Hill, NJ, USA ), with or without 250 nM PGE_2 _(Cayman Chemicals, Ann Arbor, MI, USA). Fresh IFNγ and PGE_2 _were added 24 hours later, and cells were harvested (0.25% Trypsin-EDTA) after 48 hour treatment, washed and re-suspended in FACS buffer for flow cytometry analysis.

### Bone marrow-derived macrophage isolation and culture

Bone marrow-derived macrophages (BMDM) were isolated as described [28]. Femurs from female mice were flushed with (DMEM and cells pelleted (1,000 rpm) and incubated at 37°C for 24 hours in (DMEM containing 10% FBS, 1% L-glutamine, and 1% penicillin/streptomycin. Non-adherent cells were collected and plated in L929 cell-conditioned medium (LCCM). To make LCCM, medium collected from L929 cells (American Type Culture Collection, Manassas, VA, USA), that were split 1:5 and grown to confluency, was mixed 1:5 with (DMEM with 10% FBS, 1% L-glutamine, and 1% penicillin/streptomycin. Purity (approximately 99%) was verified by flow cytometry for F4/80 and CD11b (not shown). BMDM were plated (0.5 × 10^6 ^cells/well) in LCCM. At 100% confluency, media was replaced with (DMEM. After 24 hours, cells received vehicle, or M1 polarizing mix (100 ng/ml lipopolysaccharide (LPS; Sigma) and 20 ng/ml IFNγ (Peprotech)), or M2 polarizing mix (20 ng/mL IL-4 and 10 ng/mL IL-13; Peprotech), with or without 250 nM PGE_2 _(Cayman Chemicals). Supernatants were removed 18 hours later and cells lysed for RNA isolation.

### Real Time RT-PCR

Total RNA from tumors and cells was isolated (RNeasy, Qiagen, Germantown, MD, USA), and reverse transcribed (TaqMan Reverse Transcriptase, Applied Biosystems, Carlsbad, CA, USA), according to the manufacturer's instructions. Real time quantitative (Q)-PCR of all genes, including 18S ribosomal RNA, was performed using inventoried gene expression assays and TaqMan Universal PCR Master Mix from Applied Biosystems. PCR products were detected in ABI-PRISM 7900 sequence detection systems (Applied Biosystems). Results were analyzed using the comparative Ct method, and normalized to 18S RNA.

### Immunohistochemistry

Paraffin embedded tumor tissues were sectioned (4 μm). After de-paraffinization and rehydration, endogenous peroxidase was blocked with 3% hydrogen peroxide. Heat induced epitope retrieval was performed with 1 mM EDTA (Invitrogen, Grand Island, NY, USA). After overnight blocking at 4°C with 5% donkey serum (Sigma) + 0.1% Triton 100× (Sigma), sections were incubated with primary antibodies overnight at 4°C as follows: anti-Ki67 (Abcam, Cambridge, MA, USA,1:50 dilution), anti-CD31 (Abcam, 1:200 dilution) or anti-CXCL9 (Aviva Systems Biology, San Diego, CA, USA 1:125 dilution). Thereafter, the Polink-2 HRP Plus AEC System for Immunohistochemistry (Golden Bridge International, Inc, Mukillteo, WA, USA) was used, according to the manufacturer's instructions. Slides were then counterstained with hematoxylin (Vector Laboratories, Burlingame, CA, USA), and mounted (Aqua-Mount; Lerner Laboratories, Pittsburgh, PA, USA). Images were taken with a Nikon Eclipse E600 microscope using ACT-1 imaging program (Nikon Instruments Inc., Hicksville, NY, USA).

### Flow cytometry

Single cell suspensions from tumor digestions (above) were centrifuged, washed and re-suspended in FACS buffer (PBS + 2% FBS +1 mM EDTA + 0.01% sodium azide), 1 × 10^6 ^cell/100 μl/tube. After a five minute incubation with rat anti-mouse CD16/CD32 (Mouse BD Fc Block, BD Pharmingen, Franklin Lakes, NJ, USA), 1 μg/ml of fluorescein isothiocyanate (FITC) conjugated anti-CD3, PE conjugated anti-CD4, and AF647 conjugated anti-CD8a, or PE conjugated anti-F4/80 and AF647 conjugated anti-CD86 antibodies (Invitrogen) were added. OneComp eBeads (eBioscience, San Diego, CA, USA) were incubated with anti-CD3 or anti-CD4 or anti-CD8a antibodies to perform compensation for spectral overlap. NAF COX-2KD and NAF nt (1 × 10^6 ^cell/100 μl), were incubated with PE conjugated anti-PD-L1 antibody (Biolegend, San Diego, CA USA). After a 30-minute incubation, cells were washed and re-suspended in 500 μl FACS buffer. Unstained tumor cells and cells incubated with isotype control rat anti-mouse antibody were used as negative controls. FACS analysis was performed on a BD FACSCalibur machine (BD Biosciences, San Jose, CA, USA). Data was analyzed using FlowJo Research Flow Cytometry Analysis Software (TreeStar, Ashland, OR USA).

### Orthotopic tumor growth and CD8^+ ^depletion

NAF COX-2KD and NAF nt tumor cells were injected into the #4 and #9 mammary glands (1 × 10^6 ^cells/gland in 100 μl Hanks Balanced Salt Solution) of normal WT female mice between 8 to 14 weeks of age. Orthotopic tumor volume was determined weekly using standard caliper measurement. For CD8+ depletion experiments, mice were injected intraperitoneally with 200 μg of an anti-CD8 or isotype control antibody (BioXCell, West Lebanon, NH, USA), four days and again two days prior to injection of tumor cells, and then twice weekly for a further four weeks. Depletion of CD8+ cells was confirmed by flow cytometry of erythrocyte lysed whole blood (ACK Lysing Buffer, Invitrogen), four days and again four weeks after tumor cell injections.

### Statistical analysis

Statistical analyses were performed using Prism (GraphPad Software, Inc., La Jolla, CA, USA). As appropriate, comparisons were made using logrank analysis, unpaired t-test (with Welch's correction when variances were significantly different by F-test), Mann Whitney test (when the data distribution was not normal), or, for multiple group comparisons, analysis of variance (ANOVA) followed by Bonferroni's multiple comparison test.

## Results

### Tumor onset, development, and vascularization in WT and COX-2 ^MEC^KO mice

**Figure 1:**
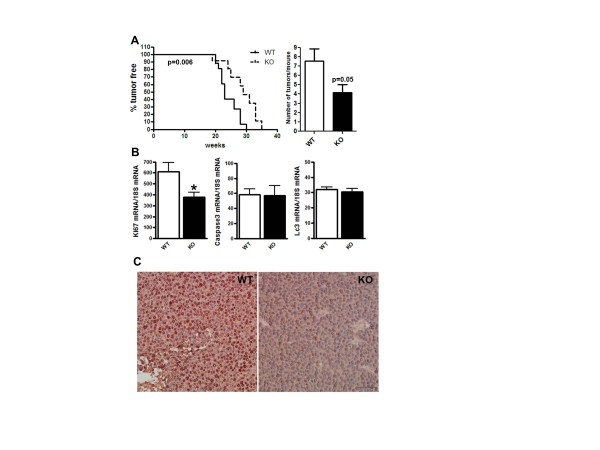
**Tumor onset, multiplicity and cell proliferation were suppressed in COX-2^MEC^KO tumors**. COX-2^MEC^KO tumors are denoted as KO. **(A) **Percent of tumor free mice against weeks of age. Mean tumor free time for COX-2^MEC^KO mice was 29 weeks versus 23 weeks for WT (left graph, *n *= 13 to 19). The right graph shows tumor multiplicity as number of tumors per mice at necroscopy (*n *= 14 to 18). **(B) **Gene expression levels of Ki67 (proliferation), Caspase3 (apoptosis) and Lc3 (autophagy) in whole tumors by Q-PCR (*n *= 8 to 18). **(C) **Immunohistochemistry staining for Ki67 (dark red-brown) in sections of paraffin embedded WT and COX-2^MEC^KO tumors (image shown is representative of *n *= 4). Cell nuclei are counterstained with hematoxylin. The bar on the KO panel indicates 20× magnification. Data in column graphs are mean ± sem. *P *values are compared to WT; *P < 0.05. COX, cyclooxygenase; KO, knock out; WT, wild type.

The current investigation was designed to study the role of MEC COX-2 in mammary tumor development, with the goal of elucidating whether and how targeted inhibition of COX-2 in epithelial cells affects the disease. In our previous study we confirmed COX-2 deletion in MEC isolated from COX-2^MEC^KO mice by Q-PCR and Western blotting, and loss of PGE_2 _generation by COX-2^MEC^KO cells was established by mass spectrometry [17]. COX-2 expression and PGE_2 _production were unchanged in peripheral macrophages isolated from COX-2^MEC^KO compared to WT confirming the selectivity of the deletion [17]. In the current study, tumor onset was significantly delayed in COX-2^MEC^KO mice compared to their WT littermates (Figure 1A). On necropsy, COX-2^MEC^KO mice had significantly fewer tumors compared to WT (Figure 1A). Consistent with these observations, cell proliferation appeared higher in WT tumors, as indicated by higher levels of mRNA for the proliferation marker Ki67 in WT compared to COX-2^MEC^KO tumors (Figure 1B). Markers for apoptosis (caspase3) and autophagy (Lc3) were not different between the two genotypes (Figure 1B). Abundant expression of Ki67 protein in WT, but not COX-2^MEC^KO, tumors was confirmed by immunohistochemistry (Figure 1C).

**Figure 2:**
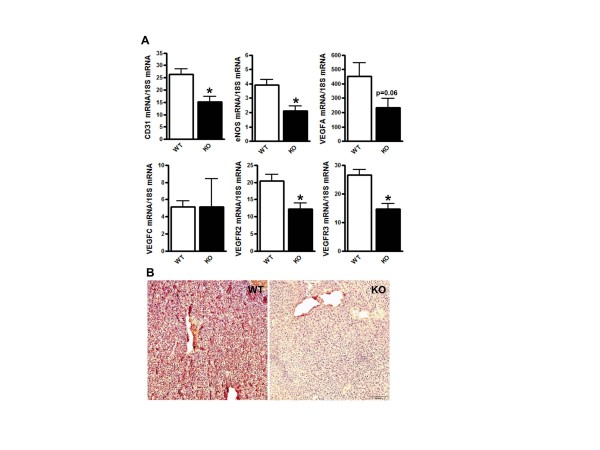
**Angiogenesis was suppressed in COX-2^MEC^KO tumors**. COX-2^MEC^KO tumors are denoted as KO. **(A) **Gene expression levels for CD31, eNOS, VEGFA, VEGFC, VEGFR2 and VEGFR3 in whole tumors by Q-PCR. Data are mean ± sem of *n *= 8 to 18. *P *values are compared to WT. *P < 0.05. **(B) **Immunohistochemistry staining for CD31 (dark red-brown) in sections of paraffin embedded WT and COX-2^MEC^KO tumors (image is representative of *n *= 6). Cell nuclei are counterstained with hematoxylin. The bar on the KO panel indicates 20× magnification. COX, cyclooxygenase; KO, knock out; WT, wild type.

Q-PCR analysis of tumors revealed lower expression levels of CD31, an endothelial marker, endothelial (e) NOS, the angiogenic factor VEGFA and its receptor VEGFR2 (Figure 2A), in COX-2^MEC^KO compared to WT. Although no difference was observed in mRNA levels of the lymphangiogenic factor VEGFC, its receptor VEGFR3 was significantly lower in COX-2^MEC^KO tumors (Figure 2A). Immunostaining for CD31 revealed a denser blood vessel network in WT tumors, confirming suppressed angiogenesis in COX-2^MEC^KO tumors (Figure 2B).

### Subpopulations and phenotypes of tumor infiltrating immune cells in WT and COX-2^MEC^KO tumors

**Figure 3:**
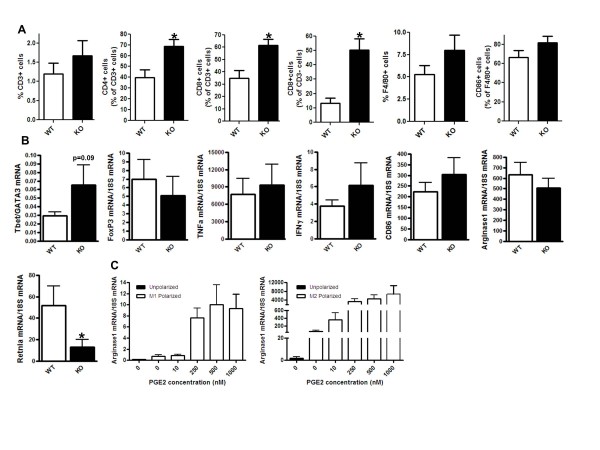
**Deletion of mammary epithelial COX-2 modified tumor infiltrating cell phenotype**. COX-2^MEC^KO tumors are denoted as KO. **(A) **Flow cytometry analysis of tumor associated lymphocytes and macrophages (*n *= 7 to 10). **(B) **Gene expression levels measured by Q-PCR in CD45^+ ^tumor infiltrating leukocytes (TILs) isolated from whole tumors by microbead separation (*n *= 6 to 7). **(C) **Gene expression levels measured by Q-PCR in unpolarized, M1 polarized or M2 polarized bone marrow derived macrophages, treated without or with PGE_2 _(*n *= 8). Data are mean ± sem. *P *values are compared to WT; *p < 0.05. COX, cyclooxygenase; KO, knock out; PGE_2_, prostaglandin E_2_; WT, wild type.

WT and COX-2^MEC^KO tumors were analyzed by flow cytometry and Q-PCR to compare the populations of infiltrating immune cells and their phenotypes. By flow cytometry, there was no difference in the total number of F4/80^+ ^TAMs between WT and COX-2^MEC^KO tumors (Figure 3A). COX-2^MEC^KO tumors did, however, have significantly higher numbers of CD3^+^CD4^+ ^cells, a population that includes T_h_1, T_h_2, and regulatory T (T_reg_) cells, as well as CD3^+^CD8^+ ^CTLs and CD3^-^CD8^+ ^cells, encompassing NK and dendritic cells (Figure 3A). To further define their functional identify, tumor-infiltrating leukocytes (TILs) were isolated using magnetic microbeads coated with a pan-leukocyte marker CD45 and cells analyzed by Q-PCR for phenotypic markers and cytokines (Figure 3B). The ratio of Tbet (T_h_1 marker)/GATA3 (T_h_2 marker) tended to be higher in COX-2^MEC^KO tumors compared to WT (Figure 3B), suggesting a prevalence of CD3^+^CD4^+ ^T_h_1 over T_h_2 lymphocytes, when MEC COX-2-derived mediators are absent. Further, mRNA levels for either Tbet alone or the Tbet/GATA3 ratio were significantly correlated with CD4 mRNA in COX-2^MEC^KO, but not WT, tumors (data not shown). Gene expression of FoxP3, a marker for T_reg_, was not altered and there was no difference in mRNA for macrophage type 1 cytokines TNFα and IFNγ or an M1 macrophage marker CD86, in CD45^+ ^TILs, suggesting no major change in M1 polarization in this disease model. COX-2-derived PGE_2 _has been implicated in driving the immune suppressive phenotype typically associated with TAM [6]. Indeed, exogenous PGE_2 _treatment significantly increased the expression of M2 marker Arginase 1, a key enzyme in suppression of T cell function, in both M1 and M2 polarized bone-marrow derived macrophages (Figure 3C). In tumors, although arginase 1 mRNA levels were similar in TILs from COX-2^MEC^KO and WT tumors, another M2 marker, Retnla, was significantly decreased in COX-2^MEC^KO (Figure 3B). Taken as a whole, our flow cytometry, immune staining and CD45^+ ^cell expression analysis indicates that absence of epithelial COX-2-derived mediators augments T_h_1 and cytotoxic immune function and reduces immune suppressive macrophage function in the mammary tumor microenvironment.

### COX-2 may enhance immune tolerance through suppression of T cell recruitment and activation

**Figure 4:**
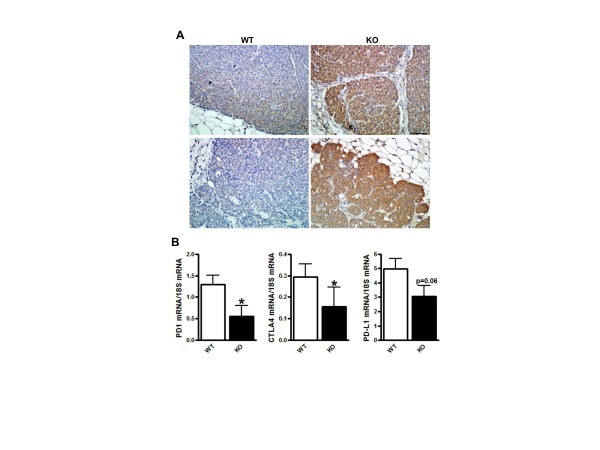
**COX-2 suppresses immune surveillance by altering T cell recruitment and activation**. COX-2^MEC^KO tumors are denoted as KO. **(A) **Immunohistochemistry staining for CXCL9 (brown) of sections from paraffin embedded WT and COX-2^MEC^KO tumors (two images from each genotype shown are representative of *n *= 4). Nuclei are counterstained with hematoxylin. The bar on the KO panel corresponds to 40× magnification. **(B) **Gene expression levels measured by Q-PCR in whole tumors (*n *= 8 to 18). Data are mean ± sem. *P *values are compared to WT; *P < 0.05. COX, cyclooxygenase; KO, knock out; WT, wild type.

**Figure 5:**
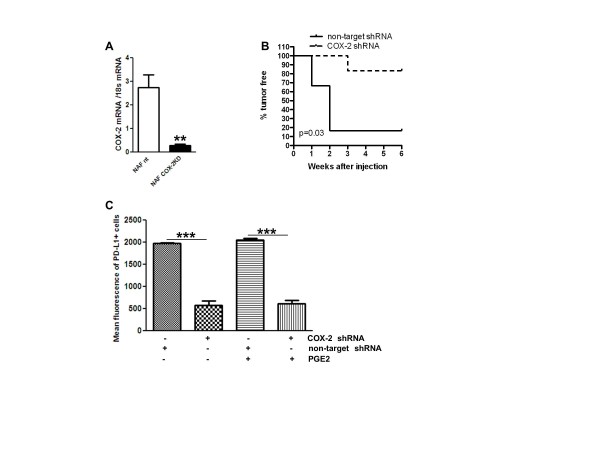
**COX-2 knock down reduced tumor cell PD-L1 expression and orthotopic tumor growth**. ErbB2-transformed mammary epithelial cells (NAF) were transduced with non-targeted shRNA (NAF nt) or COX-2 targeted shRNA (NAF COX-2KD)**. (A) **COX-2 mRNA levels measured by Q-PCR, after 24 hours of treatment with 5 μg/ml LPS (*n *= 4). **(B) **Percent of tumor free mice after mammary fat pad injection of NAF nt and NAF COX-2KD (*n *= 6). (**C) **Mean fluorescence of PD-L1 in NAF, measured by flow cytometry, after 48 hours of treatment with 10 ng/ml IFNγ, with or without 250 nM PGE_2 _(*n *= 3 to 6). Data in column graphs are mean ± sem. *P *values are compared to NAF nt control, unless otherwise indicated. *P < 0.05, **P < 0.005, ***P < 0.0005. COX, cyclooxygenase; KO, knock out; shRNA, short hairpin RNA.

Our data thus far indicates a significant contribution of mammary epithelial COX-2-derived mediators to pro-tumor immune function, particularly T lymphocyte and cytotoxic immune cell function, in the tumor microenvironment. We next examined pathways that control T cell recruitment, activation and function. In breast cancer, tumor cell expression of the chemokines CXCL9 and 10 recruits lymphocytes, improves survival in mouse models and human studies [29,30], and PGE_2 _inhibits expression of both chemokines in breast cancer cells *in vitro *[12]. Paraffin embedded sections of WT and COX-2^MEC^KO tumors showed substantially higher levels of CXCL9 expression, by immunohistochemistry, in COX-2^MEC^KO tumors, and this staining was evident throughout the tumor cells (Figure 4A). WT tumors, in contrast, showed weak CXCL9 staining (Figure 4A). T cell activation requires binding of T cell receptors to antigen and is regulated by a balance of co-stimulatory and co-inhibitory receptor-ligand interactions. T cell CD28 receptor engagement by CD80 or CD86, expressed on antigen presenting cells, provides the additional signal necessary for T cell activation. The same ligands can, alternatively, drive T cells to a state of anergy through binding to cytotoxic T lymphocyte antigen 4 (CTLA-4) [31]. Inhibition of T cell function is also directed through binding of programmed death ligand 1 (PD-L1) to its receptor, PD-1, expressed on the T cell surface [32]. In our study, gene expression levels for both inhibitory receptors CTLA4 and PD-1, as well as PD-L1, were decreased in COX-2^MEC^KO tumors compared to WT, suggesting suppressed signaling through co-inhibitory pathways (Figure 4B). Both cancer cells and tumor infiltrating myeloid cells are considered as sources of PD-L1 expression in the tumor microenvironment [33,34]. We did not observe any change in PD-L1 mRNA levels in CD45^+ ^TILs from COX-2^MEC^KO and WT tumors (data not shown), suggesting that tumor cell PD-L1 was suppressed by COX-2 deficiency. Indeed, NAF COX-2KD, which, compared to NAF nt, grew poorly as orthotopic tumors in immune competent syngenic mice (Figure 5B) also produced substantially less PD-L1 protein in response to IFNγ (Figure 5C). Interestingly, addition of exogenous PGE_2 _neither modified PD-L1 expression in NAF nt nor rescued IFNγ-induced PD-L1 expression in NAF COX-2KD cells.

**Figure 6:**
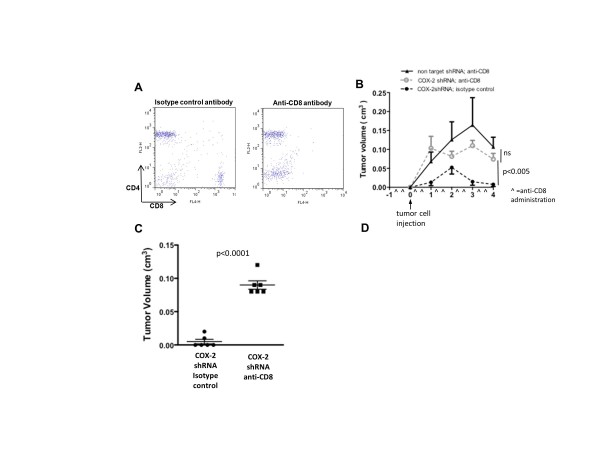
**CD8^+ ^cell depletion restores growth of injected NAF COX-2KD tumor cells as orthotopic tumors**. (A) Flow cytometry of peripheral blood from anti-CD8 or isotype control antibody treated mice four days after mammary fat pad injection (representative of *n *= 6). (B) Tumor growth of NAF nt and NAF COX-2KD cells injected into mammary fat pads of mice that received either anti-CD8 or isotype control antibody injection (antibody treatment denoted with ^ along X axis; *n *= 6). (C) NAF COX-2KD tumor volume measured on necropsy of animals that were treated with either isotype control or anti-CD8 antibody. In B and C data are mean ± sem of *n *= 6. **P < 0.005, ***P < 0.0005. COX, cyclooxygenase.

To assess how critical the loss of COX-2's immune suppressive actions was for reduced tumor growth and burden, we examined growth of NAF COX-2KD orthotopic tumors in recipient mice treated with an anti-CD8 antibody, to deplete CD8^+ ^immune cells, or an isotype control antibody. Complete depletion of CD8^+ ^cells in blood was confirmed by flow cytometry (Figure 6A). In isotype control antibody treated mice NAF COX-2KD grew poorly in only two of six injections. In contrast, six of six NAF COX-2KD tumors grew in CD8^+ ^depleted mice, similar to NAF nt control cells (Figure 6B), and were markedly larger at necroscopy (four weeks after tumor injection; Figure 6C).

## Discussion

Significant attention is now focused on understanding how resident and infiltrating cells in the tumor microenvironment support disease progression and in developing therapeutic strategies directed at microenvironmental targets [7]. Central to the pro-tumor microenvironment is suppression of immune cell function allowing tumor cells to avoid destruction. In the current study, we demonstrated enhanced immune cell recruitment and reduced T cell co-inhibitory pathways in tumors that lack mammary epithelial expression of the pro-inflammatory enzyme COX-2, coincident with delayed ErbB2 oncogene-driven mammary tumor development.

Consistent with established paradigms of COX-2 in cancer [6,35,36], deletion of MEC COX-2 delayed mammary tumor onset, lowered tumor multiplicity, reduced tumor cell proliferation and decreased tumor vascularization. Reduced vascularization in COX-2^MEC^KO tumors was associated with lower expression of VEGFA and its receptor VEGFR2, a dominant pro-angiogenic pathway in tumors [37], consistent with the role of COX-2 in promoting the angiogenic switch that allows tumors to progress [38]. It may be that reduced tumor cell proliferation and suppressed angiogenesis associated with deletion of MEC COX-2 was sufficient to suppress tumors. However, the elevation of CD4^+ ^and CD8^+ ^immune cell populations we observed in COX-2^MEC^KO mice, prompted us to consider how tumor cell COX-2 contributes to tumor immune function.

COX-2-mediated promotion of pro-tumorigenic T_h_2 lymphocyte and M2 macrophage functional phenotypes, as well as suppression of cytotoxic immune cell activity, has been reported [6]. However, it remains unclear how COX-2 contributes to the orchestration of immune cell function as tumors develop. In part, the paucity of information reflects the difficulties of working with global COX-2 knock out mice, which have breeding problems, severe renal pathology and a shortened life span [39], none of which are encountered in our targeted COX-2^MEC^KO mice, as well as the extensive use of immune deficient host mice for tumor transplant studies. Compared to WT, three populations of immune cells - CD3^+^CD4^+^, which are T_h _lymphocytes, CD3^+^CD8^+ ^cells, which are CTLs and CD3^-^CD8^+^, which encompass NKs and dendritic cells - were elevated in COX-2^MEC^KO tumors. Within the CD3^+^CD4^+ ^population, an increase in anti-tumorigenic T_h_1 cells may suppress tumors in COX-2^MEC^KO mice; however, greater activity of T_h_2 lymphocytes and/or T_reg _would be expected to promote tumor growth [24]. The strong trend towards an increased T-bet/GATA3 mRNA ratio, a measure of the T_h_1 to T_h_2 balance [40], and the unchanged expression of FoxP3, a marker for T_reg _[6,24], indicates the likely prevalence of the pro-immune helper function of T_h_1 lymphocytes over pro-tumorigenic T_h_2 lymphocytes or immune suppressive T_regs_, in COX-2^MEC^KO tumors. These data are consistent with the shift toward type 1 immunity we reported previously in carcinogen-induced mammary tumors in COX-2^MEC^KO mice, which were also delayed compared to WT [17].

Within the CD8^+ ^populations, the suppressed tumor phenotype in COX-2^MEC^KO mice may result from increased cytolytic actions of CTLs and NKs [24], as well as enhanced immunogenic actions of mature dentritic cells [41]. We did not directly discriminate between the relative contributions of these CD8^+ ^subtypes; however, a key role for CD8^+ ^immune cells in COX-2-mediated control of tumor immune function is strongly supported by the restoration of NAF COX-2KD tumor cell growth in CD8^+^-depleted mice.

TAM are abundant in mammary tumors and their density is generally directly correlated with disease severity and prognosis [42,43]. Similar to the T_h_1 and T_h_2 lymphocyte characterization, M1 and M2 macrophages are considered anti- and pro- tumor, respectively [44]. We reported previously that COX-2-derived PGE_2 _restrains M1 macrophage polarization *in vitro *and in carcinogen-induced mammary tumors [17]. In the current model, however, CD86, a M1 macrophage marker, was not different in COX-2^MEC^KO tumor associated F4/80^+ ^cells (macrophages) or in isolated CD45^+ ^TILs, compared to WT. It is likely that the relevance of COX-2-mediated paracrine control of M1 macrophage function to tumor progression varies between models. Retnla (Resistin-like molecule alpha/FIZZ1), a cytokine derived from alternatively activated M2 type macrophages [45], was significantly lower in CD45^+ ^TILs from COX-2^MEC^KO tumors suggesting reduced M2 polarization, a possible reflection of reduced T_h_2-derived cytokines in the COX-2^MEC^KO microenvironment and/or loss of paracrine COX-2-derived PGE_2 _activity, which augments M2 polarization of BMDM *in vitro*.

As a whole, our analysis of the tumor microenvironment strongly supported a shift towards enhanced helper and effector T lymphocyte recruitment and function in COX-2^MEC^KO tumors. It may be that there is simply an increased immune cell recruitment to breast tumors lacking epithelial COX-2. Indeed, we saw a dramatic increase in tumor cell expression of the T cell chemokine CXCL9 in COX-2^MEC^KO tumors, consistent with a recent report in patients with invasive breast cancer that tumor cells are the major source of CXCL9 [12]. In the same study, PGE_2 _suppressed IFNγ-induced CXCL9 levels in MCF-7 and MDA-MB 231 breast cancer cells, and COX inhibitors increased CXCL9 secretion. Despite the higher CXCL9 levels in COX-2^MEC^KO tumors, however, the absolute number of CD3^+ ^cells by flow cytometry was not higher than in WT tumors suggesting a local influence of tumor cell COX-2 derived mediators in limiting immune cell function rather than a simple recruitment effect.

Intense interest in cancer immunotherapy has focused recently on immune checkpoints, whose function to dampen immune responses is important for self tolerance and control of physiological immune responses. Two central and well-studied immune checkpoints are the co-inhibitory receptors CTLA4 and PD-1; antagonists to both are currently in clinical trials for melanoma and other cancers [32]. Engagement of CLTA4 or PD-1 on immune cells by their ligands CD80/CD86 or PD-L1, respectively, can suppress or shut down immune surveillance [46]. Conversely, blockade of co-inhibitory receptor-ligand interaction can enhance anti-tumor immunity [32]. In our study, levels of CTLA4 and PD-1, as well as PD-L1, were decreased in COX-2^MEC^KO tumors. The PD-1-PD-L1 interaction is of particular interest in this regard since PD-1 expression in tissues is induced by inflammatory signals where it acts to suppress T cell activity and limit collateral tissue damage [32]. We reasoned, therefore, that COX-2, an established inflammatory gene, may act in tumors to upregulate expression of PD-1/PD-L1, thereby suppressing immune function and facilitating immune escape. In support of this hypothesis, NAF COX-2KD, which grew very poorly as orthotopic tumors, generated substantially less PD-L1 in response to IFNγ compared to NAF nt control cells. The failure of exogenous PGE_2 _to restore PD-L1 expression levels in NAF COX-2KD may suggest distinct actions of autocrine and paracrine PGE_2_, or indicate a role for other COX-2-derived products, in tumor cell COX-2 mediated control of PD-1 expression. The pathways through which COX-2-derived PGE_2_/other prostanoids control tumor cell expression of PD-L1 and other immune modulators are currently under investigation.

Our study provides significant insight into the complex autocrine and paracrine functions of mammary epithelial COX-2 in ErbB2-induced breast cancer and suggests that tumor cell COX-2 is an important component in establishing a permissive immune microenvironment. Recent studies indicated that CD8^+ ^tumor infiltration bolstered chemotherapeutic responses in human breast cancer and mouse models [8]. Our demonstration that deletion of tumor cell COX-2 can enhance tumor-associated CD8^+ ^cytotoxic immune cell infiltration and function may open new avenues to develop targeted strategies for COX-2 inhibition in combination with cytotoxic drugs. Further, there have been significant advances in cancer immunotherapy using antibodies to block CTLA4 or PD-1 co-inhibitory function, thereby augmenting anti-tumor immunity [32,47-49]. To our knowledge, our study is the first to link COX-2 to T cell co-inhibitory receptor/ligand function, a potentially new avenue to investigate COX-2 inhibitors as adjuvants to immunotherapy. Finally, we demonstrated that interruption of COX-2 function selectively in epithelial cells was sufficient to reduce ErbB2- (this study) and carcinogen [17] induced mammary tumorigenesis and growth. The clinical use of systemic COX-2 inhibitors in cancer, although supported across multiple studies [1], is limited by the associated gastrointestinal and cardiovascular hazards [50]. We speculate that, as improved targeted drug delivery modalities continue to emerge, delivery of COX-2 selective inhibitors directly to the tumor cells may allow for safe and effective use of these drugs in cancer without the deleterious side effects associated with systemic COX inhibition.

## Conclusions

The data strongly support that, in addition to its angiogenic function, tumor cell COX-2-derived mediators suppress anti-tumor immune cell function, possibly through upregulation of inhibitory immune checkpoints, contributing to tumor immune escape. COX-2 inhibition may be clinically useful to augment breast cancer immunotherapy.

## Abbreviations

BMDM: bone marrow derived macrophages; BSA: bovine serum albumin; COX: cycloogygenase; CTL: cytotoxic T lymphocyte; CTLA-4: cytotoxic T lymphocyte antigen 4; (D)MEM: (Dulbecco's) modified Eagle's medium; EDTA: ethylenediaminetetraacetic acid; FACS: fluorescence-activated cell sorting; FBS: fetal bovine serum; IFN: interferon; KO: knock out; KD: knock down; MEC: mammary epithelial cell; mmtv: mouse mammary tumor virus; NK: natural killer cells; nt: non-target; PCR: polymerase chain reaction; PD-1: programed death 1; PD-L1: programmed death ligand 1; PG: prostaglandin; shRNA: small hairpin RNA; TAM: tumor associated macrophage; TIL: tumor infiltrating leukocyte; TNF-α: tumor necrosis factor-α; WT: wild type.

## Competing interests

The authors declare that they have no competing interests.

## Authors' contributions

NM made substantial contributions to the study design, data acquisition, analysis and interpretation, as well as the writing and editing, of the manuscript. EPC contributed to data acquisition and analysis in the macrophage and flow cytometry experiments. RAE contributed to the design of the immune depletion experiments. VN contributed to the data acquisition. RHV contributed to the analysis and interpretation of the immune function data. EMS directed the conception and design of the study and the experimental work, contributed to the analysis and interpretation of data and critically revised the manuscript for important intellectual content. All authors read and approved the final manuscript.
